# Anti-CTLA-and anti-PD-1 immune checkpoint inhibitor antibodies impair human sperm motility *in-vitro*

**DOI:** 10.3389/fphar.2025.1534975

**Published:** 2025-03-31

**Authors:** Ilaria Cosci, Luca De Toni, Paolo Del Fiore, Andrea Di Nisio, Samuela Carraro, Claudia Maria Radu, Loris Bertazza, Simone Mocellin, Jacopo Pigozzo, Giovanna Crivellaro, Marina Coppola, Alberto Ferlin

**Affiliations:** ^1^ Department of Medicine, Unit of Andrology and Reproductive Medicine, University of Padova, Padova, Italy; ^2^ Soft-Tissue, Peritoneum and Melanoma Surgical Oncology Unit, Veneto Institute of Oncology IOV-IRCCS, Padova, Italy; ^3^ Department of Health, Nutrition and Sport, Pegaso Telematic University, Naples, Italy; ^4^ Department of Medicine, Hematology and Clinical Immunology Branch, University of Padova, Padova, Italy; ^5^ Department of Medicine, Thrombotic and Haemorrhagic Disease Unit and Haemophilia Center, University of Padova, Padova, Italy; ^6^ Departiment of Medicine, Unit of Endocrinology, University of Padova, Padova, Italy; ^7^ Department of Surgical, Oncological and Gastroenterological Sciences (DISCOG), University of Padova, Padova, Italy; ^8^ Medical Oncology 2, Veneto Institute of Oncology IOV-IRCCS, Padua, Italy; ^9^ Pharmacy Unit, Veneto Institute of Oncology IOV-IRCCS, Padova, Italy; ^10^ IOV, Istituto Oncologico Veneto, IRCCS, Padova, Italy

**Keywords:** ipilimumab, Nivolumab, Pembrolizumab, Annexin-V, TUNEL

## Abstract

**Background:**

Immune checkpoint inhibitors (ICIs), namely, anti-cytotoxic T lymphocyte-associated protein 4 (CTLA-4) monoclonal antibody Ipilimumab and anti- and programmed cell death 1 (PD-1) monoclonal antibodies Nivolumab, and Pembrolizumab, have improved the treatment outcomes for many other cancer types. However, their impact on fertility remains under-explored.

**Methods:**

The possible direct effects of ICIs on human sperm was investigated. Spermatozoa from ten normozoospermic donors were exposed to Ipilimumab, Nivolumab, or Pembrolizumab at concentrations ranging from 1 to 100 ng/mL. Sperm motility was assessed through standard laboratory process. Cell viability and apoptosis markers were evaluated by flow-cytometry using fluorescent Annexin-V probe and Terminal Uridine Nick-End Label (TUNEL) assays. Protein-A-purified therapeutic antibodies (IgG) were also evaluated.

**Results:**

Spermatozoa had high PD-1 (>99%) and negligible CTLA-4 expression. Exposure to ICIs, was associated with a concentration-dependent impairment of sperm motility, noticeable for Pembrolizumab and Ipilimumab since 10 ng/mL, and for Nivolumab since 100 ng/mL. However, no significant effect on cell apoptosis or viability was shown. Purified IgG from ICIs maintained the adverse effect on cell motility without affecting viability.

**Conclusion:**

ICIs, specifically Pembrolizumab, Nivolumab, and Ipilimumab, adversely affect human sperm motility *in vitro*. Further research is required to understand the underlying mechanisms and clinical implications.

## Introduction

Advancements in cancer treatment over the years have improved long-term prognoses and remission of the disease (Chabner and Roberts, 2005). However, since conventional treatments such as chemotherapy and radiotherapy target rapidly dividing cells, including both cancer cells and healthy high-turnover cells in the body, they are associated with severe cellular damage in rapidly renewing tissues (Kuderer et al., 2022). This is particularly the case of the seminiferous tubule within the testis, where spermatogenesis takes place which is highly vulnerable to the damage associated with these therapies (Jensen et al., 2022). Accordingly, fertility preservation in men undergoing chemotherapy or radiotherapy involves sperm cryopreservation, a well-established clinical protocol endorsed by most international guidelines on comprehensive cancer care (Gao et al., 2019).

The advent of the immunotherapy approach to cancer treatment has revolutionized the management of several cancers, offering hope to patients with previously limited therapeutic options. In general, cancer immunotherapy involves different mechanistic strategies, such as activating innate and adaptive anti-cancer immunity or abrogating cancer-dependent immune-suppressive processes, such as immune checkpoint inhibitors (ICIs) (Sharma et al., 2011). In this context, two key molecular drivers of cancer immune-suppression have been identified: the cytotoxic T lymphocyte-associated protein 4 (CTLA4) and programmed cell death 1 (PD1) (Sharma et al., 2011). CTLA4 exerts immunosuppression by reducing CD28-mediated signaling through direct out-competition with CD28, to which it is much more similar than the CD28-stimulatory receptors CD80 and CD86, and by inducing the membrane downregulation of CD80 and CD86 themselves (Rudd et al., 2009; Qureshi et al., 2011). On the other hand, PD1 is involved in immune-response containment of tumor-specific T cells and lymphocytes upon activation by PD1-ligand 1 (PD1-L1) expressing-tumor cells or tumor infiltrating-immune cells (Honda et al., 2017). On this basis, the anti-CTLA-4 blocking antibody Ipilimumab and the anti PD-1-blocking antibodies Nivolumab and Pembrolizumab have shown clinical efficacy in the treatment of, respectively, melanoma, small and non-small cell lung cancer, renal cell carcinoma and Hodgkin’s lymphoma (Ansell et al., 2015; Borghaei et al., 2015; Motzer et al., 2015; Robert et al., 2015; Ribas et al., 2016; Rittmeyer et al., 2017).

On the other hand, it has recently been reported that ICIs are associated with altered immune responses and inhibition of T cell activity, resulting in a spectrum of inflammatory side effects known as immune-related adverse events (Michot et al., 2016). As a result, more than half of patients experience side effects involving multiple organs, including systemic immune-inflammatory syndrome and, in particular, endocrinopathies (Lu et al., 2019). In this context, the impact of ICIs on the reproductive system, particularly male fertility, remains uncertain. Notably, spermatozoa-targeting autoimmunity, such as the development of anti-sperm antibodies, severely impacts male fertility through various mechanisms, mostly by diminishing sperm motility (Vickram et al., 2019). Despite sporadic case reports exhibiting methodological limitations and conflicting findings, comprehensive studies exploring the gonadal toxicity of Ipilimumab, Nivolumab, and Pembrolizumab are scarse (Rabinowitz et al., 2021a).

The aim of this study was to explore the possible direct effect of Ipilimumab, Nivolumab, and Pembrolizumab on human spermatazoa *in vitro*. We focused in particular on the functional correlates to ICIs exposure, such as the impact on cell motility, viability and triggering of apoptotic events.

## Methods

### Reagents

Nivolumab, Ipilimubab and Pembrolizumab were kindly provided by the Pharmacy Unit of the Istituto Oncologico Veneto IRCCS (IOV), as medicinal specialties leftover from their clinical use. Sperm washing medium (SWM), a CE marked, sterile, iso-osmolar culture medium used for the standard processing of human spermatozoa, was purchased from FUJIFILM Europe (Tilburg, Netherlands). Cell Death Detection Kit and DNAse-I were purchased from Roche Diagnostics (Milan, Italy). Triton X-100, propidium iodide (PI) and Phytohemagglutinin (PHA) were purchased from Merck-Sigma (Milan, Italy). Allophycocyanin (APC)-conjugated mouse Anti-Human CD152 (Cat.#569655, CTLA-4), Phycoerythrin (PE)-conjugated mouse Anti-Human CD279 (cat# 569465, PD-1), Fluorescein-isothiocyanate (FITC)-labelled Annexin-V (Cat.# 556420) and 10× Annexin-V reaction Buffer (Cat.# 556454) were all purchased from BD Biosciences (Milan, Italy). Protein A-IgG Purification Kit (Cat.# 44667) was purchased from Life Technologies (Milan, Italy).

### Semen samples

The study was approved by the Territorial Ethics Committee Central-Eastern Veneto Area, Italy, (protocol number AOP2745). The investigation was performed according to the principles of the Declaration of Helsinki. Semen samples were collected from 10 normozoospermic patients with proven fertility. All patients (age range 20–45 years) attended the Unit of Andrology and Reproductive Medicine of the University-Hospital of Padova. Upon signing a dedicated written informed consent form at the first outpatient evaluation, patients were then consecutively enrolled. Inclusion criteria for donors included: age between 20 and 35 years, no history of reproductive diseases, and normal semen parameters according to World Health Organization (WHO) criteria (WHO laboratory manual for the examination and processing of human semen Sixth Edition, 2020). The semen samples were obtained by masturbation after 2-5 of sexual abstinence days and were collected in sterile containers. After allowing liquefaction for 30 min at 37°C, samples were examined for sperm parameters according to the WHO criteria (WHO, 2021). All samples showed normal semen volume, viscosity, pH, leukocyte count, negative microbiological testing and absence of anti-sperm antibodies. Medical history confirmed the absence of known genetic causes of infertility, obstructive azoospermia, varicocele, testicular masses screened by scrotal color Doppler ultrasonography, orchitis, testicular torsion/trauma, current or previous cancer disease and use of drugs with acknowledged gonadal toxicity for all patients.

### Sperm processing

Experimental procedures on naïve spermatozoa were performed upon cell washing in SWM and centrifugation for 5 min at 1,200 × g. Motile spermatozoa were isolated by swim-up selection (WHO laboratory manual for the examination and processing of human semen Sixth Edition, 2020). Briefly, after liquefaction, 1 mL of semen was layered under 1 mL of SWM and incubated at 37°C in a 5% CO_2_ atmosphere for 1 h. Motile spermatozoa that migrated into the overlying SWM were collected and used for subsequent analyses. Subsequently, samples of either naïve or swim-up selected spermatozoa were properly aliquoted, incubated with the different concentrations of Ipilimumab, Nivolumab or Pembrolizumab at 37°C in a 5% CO_2_ atmosphere for up to 4 h in agreement with previous studies using therapeutic monoclonal antibodies on human spermatozoa *in vitro*. Samples were then assessed as described below. In the control condition (CTRL), immunotherapeutic agents were omitted.

### TUNEL test, Annexin-V test and phenotypic characterization analysis

Spermatozoa were fixed in 4% paraformaldehyde-PBS solution for 15 min at room temperature, washed in phosphate-buffered saline (PBS) and centrifuged at 1,200 × g for 5 min at room temperature. The cell pellet was then resuspended and permeabilized in 200 μL of 0.1% Triton X-100 in 0.1% sodium citrate solution for 4 min at 2°C–8°C. Samples were then washed in 0.1% bovine serum albumin (BSA)-PBS solution and centrifuged at room temperature for 5 min at 1,200 × g. DNA fragmentation was evaluated by Terminal Transferase dUTP Nick End Labeling (TUNEL) assay performed by Cell Death Detection Kit according to the manufacturer’s instructions. Cells were counterstained with 25 ng/mL propidium iodide solution in order to distinguish permeabilized spermatozoa. Positive controls were obtained by incubating cells with 1 g/mL DNAse I for 45 min at 37°C.

Early cell apoptosis analysis was performed by Annexin V-FITC Apoptosis Detection Kit according to the manufacturer’s instructions. Briefly, approximately 2 × 10^6^ cells per sample were resuspended in 500 µL of 1× Annexin-V Reaction Buffer. Subsequently, 5 µL of Annexin V-FITC and 5 µL of propidium iodide solutions were added to the cell suspension and left to incubate for 15 min at room temperature in the dark. Cells were then evaluated with a benchtop CytoFLEX flow cytometer platform and data analyzed with CytExpert SRT software (Beckman-Coulter, Rome, Italy).

Phenotypic characterization of human spermatozoa for CTLA-4 and PD-1 expression was assessed by direct incubation of washed naïve spermatozoa with APC-conjugated anti CTLA-4 and PE-conjugated anti PD-1 antibodies (5 μL each) for 20 min at room temperature in the dark. Freshly prepared peripheral blood mononuclear cells (PBMC), eventually stimulated with 20 μg/mL PHA for 3 days, were used as reference and evaluated by FACS-Canto II Flow cytometer (BD Biosciences, Milan, Italy). Localization of CTLA.4 and PD-1 antigens on cells was performed using the Amnis ImageStream Mk II Imaging flow cytometer (Luminex, Thermo Fisher Scientific). Data were acquired with INSPIRE software and analyzed using the IDEAS 6.3 analysis software (Luminex, Thermo Fisher Scientific).

### Statistical analysis

Statistical analyses were performed using SPSS 23 Statistics for Windows (Chicago, IL) and GraphPad PRISM 9.4.0 (GraphPad Software, San Diego, CA, United States). The results were expressed as means ± standard deviation (SD). The Kolmogorov-Smirnov test was used to verify normal distribution of continuous variables. The comparison of the mean values of two groups was performed by Student’s t-test. Multivariate analysis with Bonferroni’s *post hoc* correction was used to evaluate the effect of the interaction between the specific ICC and its concentration on cellular function. Non-parametric Mann Whitney U test was used to compare two groups of continuous variables with non-normal distribution. Values of P < 0.05 were considered significant.

## Results

### Phenotypic characterization of spermatazoa for CTLA-4 and PD-1 expression

**FIGURE 1:**
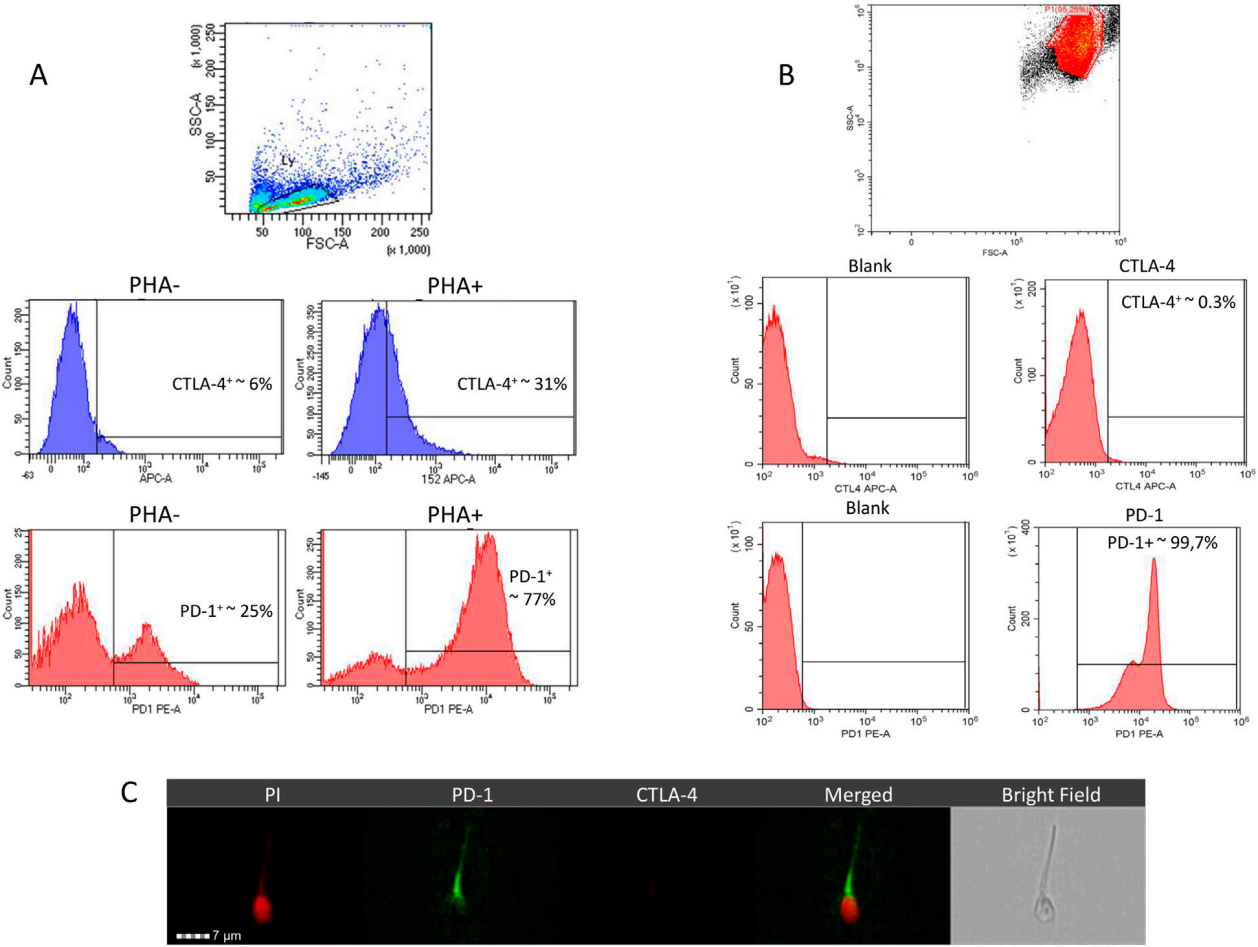
Representative images of flow-cytometry analysis for the phenotypic characterization, in peripheral blood mononucelar cells (PBMC) **(A)** and in human spermatozoa **(B)**, of anti-cytotoxic T lymphocyte-associated protein 4 (CTLA-4) monoclonal and anti- and programmed cell death 1 (PD-1) expression. Expression of CTLA-4 and PD-1 in PBMC was evaluated in both naïve (PHA-) and upon stimulation with Phytohemagglutinin (PHA+) for 3 days. **(C)** Representative images of CTLA-4 and PD-1 localization in human sperm cells evaluated by immunofluorescence through imaging flow cytometer. Cell nucleus was counterstained with propidium iodide (PI).

In order to address the phenotypic characterization of human spermatazoa for CTLA-4 and PD-1 by labelled primary antibody immunostaining, assay antibodies were formerly validated on peripheral blood mononuclear cells (PBMC) at baseline and after stimulation with 20 μg/mL PHA for 3 days as previously described (Gribben et al., 1995) (Figure 1A). Compared to a baseline expression of CTLA-4 in approximately 6.1% ± 3.2% of PBMC, PHA stimulation was associated with a significant increase in stained cells up to 31.4% ± 7.9% (P = 0.0068 vs. baseline). In parallel, baseline expression of PD-1 in 24.9% ± 9.6% of PBMC showed a massive increase to 77.3% ± 10.4% (P = 0.0030 vs. baseline) upon stimulation with PHA, confirming the reliability of assay antibodies.

Evaluation of CTLA-4 and PD-1 expression in freshly ejaculated human spermatozoa showed massive expression of PD-1 (>99%) in the whole cell population but CTLA-4 was essentially undetectable (Figure 1B). To confirm this evidence, cell localization of CTLA-4 and PD-1 was assessed by imaging flow cytometry (Figure 1C). Image analysis showed a clear signal for PD-1 immune-staining on cell surface at neck and tail regions, clearly surrounding propidium iodide (PI)-based nuclear staining of cell nucleus. Conversely, immunostaining for CTLA-4 was confirmed to be negative.

### Effect of exposure to ICIs on sperm function

**FIGURE 2:**
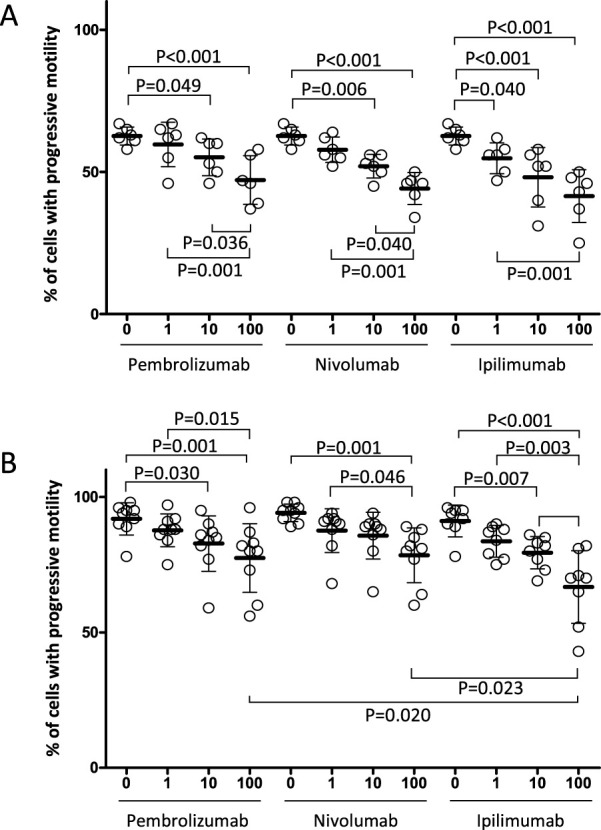
Effect of the *in vitro* exposure to Nivolumab, Ipilimubab or Pembrolizumab, of human spermatozoa from freshly ejaculated semen **(A)** or motile sperm fraction isolated by swim up selection **(B)**. Cells were exposed to drugs for 4 h at 37°C at the concentrations indicated. Singular results and mean values ± standard deviations are reported. Significance: P values for the comparison of single conditions are reported.

In order to investigate the possible functional correlates associated with direct exposure to ICIs, human spermatazoa from whole semen were treated *in vitro* with medicinal specialties containing Pembrolizumab, Nivolumab or Ipilimumab for 4 h, all at concentrations ranging from 1 to 100 ng/mL (Figure 2A). These concentrations were chosen according to available data on serum levels of ICIs in patients receiving drugs for treatment purposes (de Jong et al., 2022). Proper dilution factors were then applied in order to simulate a likely level of diffusion into seminal plasma as previously described for other therapeutic antibodies (Sohn et al., 2016). Compared to untreated controls, treatment with Pembrolizumab or Nivolumab was associated with a significant and progressive decrease in the percentage of spermatozoa with a progressive motility pattern, at a concentration equal to or greater than 10 ng/mL. For Ipilimumab, a significant decrease in the sperm motility parameter was observed even at the lowest concentration tested of 1 ng/mL. Importantly, no specific differential effects for one drug over the others were observed. In order to remove any possible background effect on spermatazoa at ejaculation, an analogous experiment was performed on spermatozoa isolated based on high motility characteristics using the swim-up technique (WHO laboratory manual for the examination and processing of human semen Sixth Edition, 2020). A similar decreasing pattern of sperm progressive motility was observed along with the increase in ICIs concentration in sperm culture media (Figure 2B). In particular, for Pembrolizumab and Ipilimumab, a significant and progressive decrease of in motility was observed from exposure to concentrations equal to or greater than 10 ng/mL whilst Nivolumab caused a significant reduction in the motility pattern only at the highest concentration of 100 ng/mL. Once again, no specific differential effects for one drug over the others were observed.

**FIGURE 3:**
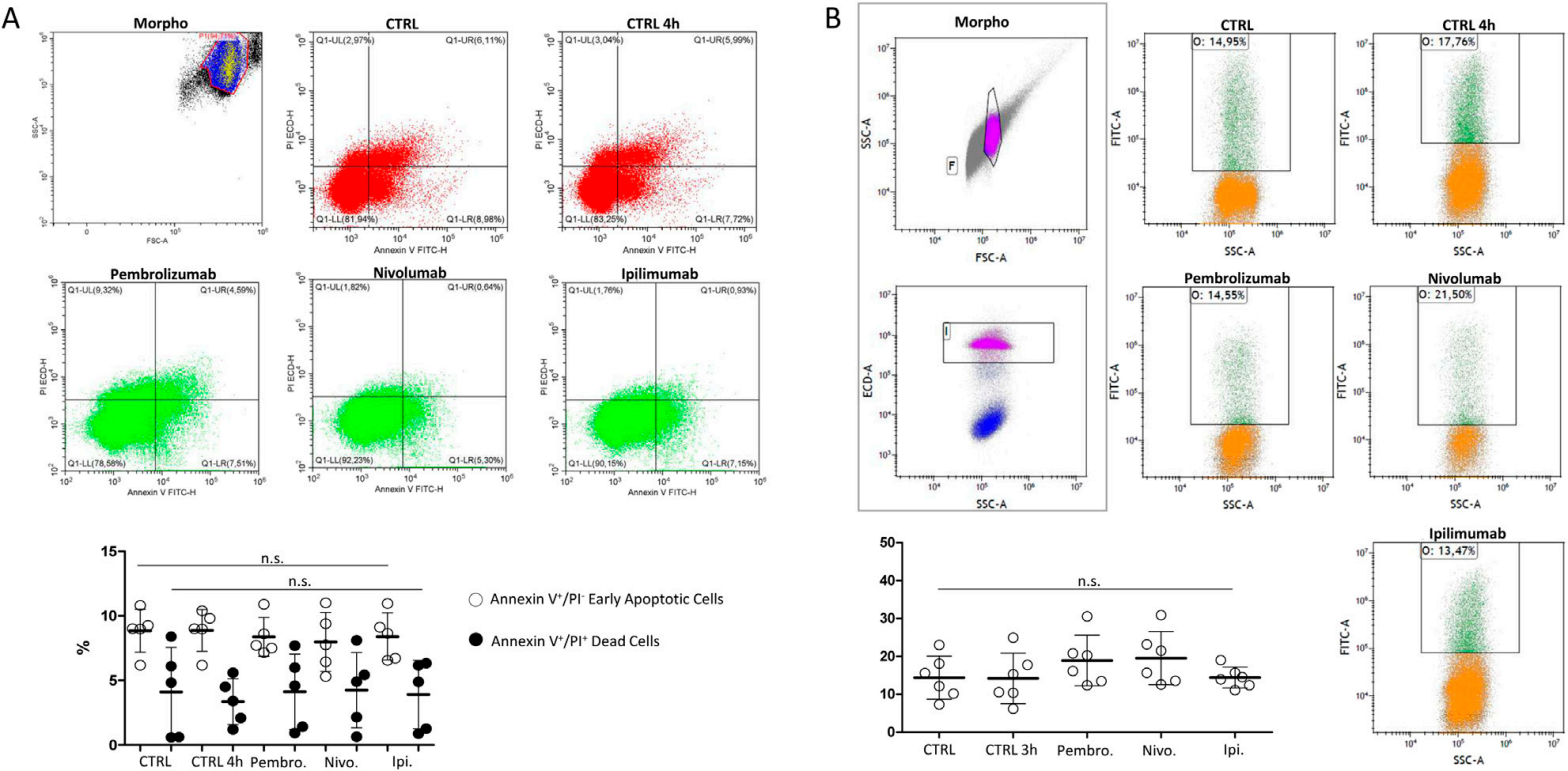
**(A)** Flow-cytometry evaluation of sperm cell apoptosis by Annexin-V staining/propidium iodide (PI) counterstaining exposed to immune checkpoint inhibitors Pembrolizumab, Nivolumab or Ipilimumab for 4 h at 37°Cs. In control condition (CTRL), drugs were omitted. Single positive Annexin V^+^/PI^−^ events were considered as early apoptotic cells. Double positive Annexin V^+^/PI^+^ events were considered as dead cells. **(B)** Flow-cytometry evaluation of DNA fragmentation by Terminal Uridine Nick-End Label (TUNEL). Cells were counterstained with PI and double positive TUNEL^+^/PI^+^ events were considered as cells with fragmented DNA. Representative images of the morphological gating strategies (Morpho) are reported. Scatterplots of experimental replicates show the quantitative comparisons among conditions. Significance: n.s. = not significant among the indicated conditions.

In order to address whether the observed effect on sperm motility was associated with the impairment of cell viability, the effect of ICIs exposure on early and late cell apoptosis events was investigated, on isolated spermatozoa from freshly ejaculated semen, at the highest concentration of 100 ng/mL for each drug. In particular, staining to annexin-V and TUNEL test were used to observe early membrane phosphatidyl-serine externalization and DNA fragmentation, respectively (Garolla et al., 2015) (Figures 3A, B). Compared to the untreated control, 4-hour exposure to ICIs showed no significant variation on the percentage of annexin-V-positive apoptotic cells, annexin-V-positive/PI double positive dead cells or TUNEL-positive cells with fragmented DNA (all P > 0.05).

### Effect of the exposure to purified therapeutic antibodies on sperm function

**FIGURE 4:**
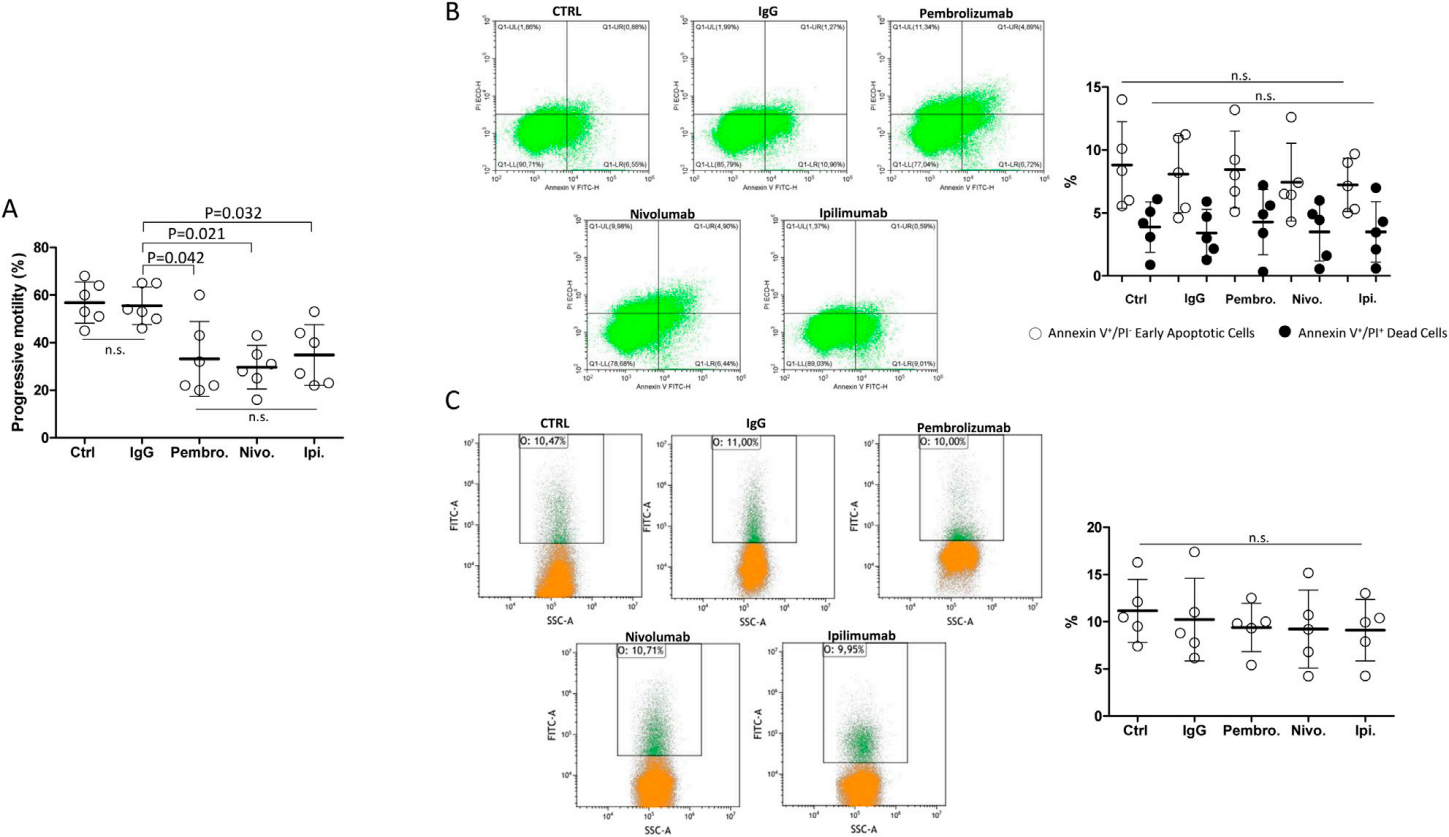
**(A)** Effect of the *in vitro* exposure to terapeutic antibodies isolated from Nivolumab, Ipilimubab or Pembrolizumab, isolated by protein-A approach from medicinal specialties, on human spermatozoa from freshly ejaculated semen. Class G Immunoglobulins (IgG) from peripheral blood were used as reference. Cells were exposed to drugs for 4 h at 37°C at the concentrations indicated. Singular results and mean values ± standard deviations are reported. Significance: P values for the comparison of single conditions are reported; n.s. = not significant. **(B)** Flow-cytometry evaluation of sperm cell apoptosis by Annexin-V staining/propidium iodide (PI) counterstaining exposed to purified immunoglobulins for 4 h at 37°Cs. In control condition (CTRL), drugs were omitted. Single positive Annexin V^+^/PI^−^ events were considered as early apoptotic cells. Double positive Annexin V^+^/PI^+^ events were considered as dead cells. **(C)** Flow-cytometry evaluation of DNA fragmentation by Terminal Uridine Nick-End Label (TUNEL). Cells were counterstained with PI and double positive TUNEL^+^/PI^+^ events were considered as cells with fragmented DNA. Representative images of the morphological gating strategies (Morpho) are reported. Scatterplots of experimental replicates show the quantitative comparisons among conditions. Significance: n.s. = not significant among the indicated conditions.

In order to address whether the observed effect of ICIs on sperm function was related to the antibody function or the excipient fraction of the medicinal specialty, the therapeutic IgG fraction from medicines containing Pembrolizumab, Nivolumab or Ipilimumab was purified by a protein A isolation approach, as previously described, and tested on isolated spermatozoa from freshly ejaculated semen (Goding, 1978). Unrelated IgG were obtained from healthy blood donors as control antibodies. Compared to either untreated control or unrelated IgG, purified Pembrolizumab, Nivolumab and Ipilimumab antibodies, all tested at the highest concentration of 100 ng/mL, significantly reduced the cell motility parameter in washed spermatozoa from healthy donors with no specific differential effects for one drug over the others (Figure 4A). However, the investigation on early and late cell apoptosis events, evaluated by the percentage of annexin-V-positive apoptotic cells, annexin-V-positive/PI double positive dead cells or TUNEL-positive cells with fragmented DNA, showed no significant difference compared to either the untreated control or unrelated IgG (Figures 4B, C).

## Discussion

In this study we provide evidence, for the first time to the best of our knowledge, that therapeutic monoclonal antibodies Pembrolizumab, Nivolumab and Ipilimumab, commonly used as immune checkpoint inhibitors, have detrimental effects on cell motility in human spermatozoa *in vitro*. Furthermore, this effect appears to be unrelated to the expression of the specific molecular target of ICIs by spermatozoa.

The reproductive outcomes associated with the use of therapeutic monoclonal antibodies is a current matter of debate. In a recent study by Handelsman et al., the longitudinal evaluation of semen parameters was evaluated in patients receiving non-cytotoxic and immunotherapy (NCIT) drugs, including immunotherapy checkpoint or enzyme inhibitors or analogous non-cytotoxic drugs (Handelsman et al., 2024). Particular attention was given to two groups of patients: men scheduled to receive NCIT drugs with no previous cytotoxic drugs or radiotherapy treatment and patients already receiving NCIT. Overall, NCIT treatment was associated with a significant reduction in sperm count and concentration independently from any covariate considered, including single drugs or their associations. Importantly, the concomitant increase in follicle stimulating hormone (FSH) levels suggested a direct testicular toxicity (Handelsman et al., 2024). Specifically dealing with immune checkpoint inhibitors, an overview of current knowledge on this topic is well described in a review by Garutti et al. (Garutti et al., 2021). Although hypophysitis is reported in 5.6% of patients receiving anti-CTLA4 antibodies and in 0.5%–1.1% of patients receiving anti-PD1 antibodies, hypogonadism is rarely described (Ryder et al., 2014; Barroso-Sousa et al., 2018). Conversely, direct damage to testicular function appears to be much more consistent, resulting in various degrees of reduced sperm count to azoospermia, alone or associated with orchitis (Brunet-Possenti et al., 2017; Quach et al., 2019; Scovell et al., 2020; Rabinowitz et al., 2021b; Salzmann et al., 2021). However, the actual epidemiological extent and causal relationships are difficult to establish given the occasional nature of the reports and the presence of numerous confounding factors (Garutti et al., 2021).

Our results strongly support the possible direct toxicity of therapeutic antibody-based ICIs, as human spermatozoa exposed *in vitro* to either Ipilimumab, Nivolumab or Pembrolizumab show a significant and dose-dependent impairment of the motility function. Importantly, this effect appears to be specific as it is consistent in the naïve population and in the viable population selected for motility. In addition, it is not associated with processes of compromised cell viability. Unfortunately, we are currently unable to explain why a target-specific effect cannot be detected even though human spermatozoa express PD-1 only. One possible hypothesis might involve the interaction of humanized antibodies ICIs with the Fc receptor on spermatozoa, resulting in compromised motility (Kamada et al., 1991). However, since unrelated IgG from donor sera had essentially no effect on cell motility, the contribution of this mechanism appears as negligible. Differently, an anti-sperm antibody (ASA)-like mechanism can be invoked. ASA are clinical findings frequently detected during infections of the male genital tract, are involved in the anti-pathogen immune reaction and are associated with the impairment of semen parameters, including motility. The mechanisms underlying this evidence have not been clarified but are believed to be linked to the interference with the energy metabolism of the cell without involving DNA fragmentation (Chenafi-Adham et al., 2024).

Whether and to what extent our findings translate into an actual clinical risk of infertility is a matter of investigation. Differently from cytotoxic chemotherapy which has been associated with an overall impairment of spermatogenesis, from a reduced sperm count to altered cell motility and sperm DNA fragmentation, our data suggest that ICIs essentially affect sperm motility. Thus, since motility is a key function involved in for natural fertility, necessary to allow the proper interaction between the oocyte and spermatozoa, significant impact on the chances of spontaneous conception with an increasing access to assisted reproductive techniques (ART) should be expected (Gaddum-Rosse, 1981). However, additional longitudinal studies are required to address the possible association between the use of ICIs and reduced fertility potential.

We acknowledge the reduced sample size as a major limitation of the study. However, although ancillary, this evidence is likely to have clinical relevance, as the immunoglobulin content of seminal plasma is essentially of transudative origin from the circulation.

## Data Availability

The raw data supporting the conclusions of this article will be made available by the authors, without undue reservation.
